# Efficacy of a novel chewable tablet (Credelio Quattro^™^) containing lotilaner, moxidectin, praziquantel, and pyrantel for the treatment and control of *Echinococcus multilocularis* and *E. granulosus* infections in dogs

**DOI:** 10.1186/s13071-025-06735-w

**Published:** 2025-03-11

**Authors:** Samuel Charles, Gertraut Altreuther, Xinshuo Wang, Scott Wiseman, Craig R. Reinemeyer, Daniel E. Snyder, Michael Ulrich, Padraig Doherty, Lisa Young

**Affiliations:** 1 Elanco Animal Health, 2500 Innovation Way, Greenfield, IN 46140 USA; 2 Elanco Animal Health GmbH, Alfred-Nobel-Str. 50, 40789 Monheim, Germany; 3 Elanco Animal Health, Form 2, Bartley Way, Bartley Wood Business Park, Hook, RG27 9XA UK; 4 East Tennessee Clinical Research, Inc, 80 Copper Ridge Farm Road, Rockwood, TN 37854 USA; 5 Daniel E. Snyder DVM, Ph.D. Consulting LLC, 7348 Yorkshire Blvd., Indianapolis, IN 46229 USA; 6 Cheri-Hill Kennels, 17190 Polk Rd., Stanwood, MI 49346 USA; 7 Iorras Product Development Ltd., Glenamoy, Ballina Co. Mayo Ireland

**Keywords:** Praziquantel, Efficacy, Cestode, *Echinococcus granulosus*, *Echinococcus multilocularis*

## Abstract

**Background:**

While generally harmless to dogs, the cestode species *Echinococcus granulosus* and *Echinococcus multilocularis* have significant zoonotic importance, causing cystic echinococcosis and alveolar echinococcosis in humans, respectively. Regular deworming is essential to treat intestinal cestode infections in dogs and to reduce environmental egg contamination and thus the subsequent zoonotic risk of infection to intermediate hosts and humans. The studies described here evaluated the efficacy of a new novel chewable tablet combination containing lotilaner, moxidectin, praziquantel and pyrantel (Credelio Quattro, Elanco Animal Health) against *E. granulosus* and *E. multilocularis* infections in dogs.

**Methods:**

Four negative-controlled, masked, randomized laboratory studies were conducted. Two studies each evaluated the efficacy of Credelio Quattro at the minimum dosages of 20.0 mg/kg lotilaner, 0.02 mg/kg moxidectin, 5.0 mg/kg praziquantel and 5.0 mg/kg pyrantel against *E. granulosus* (Studies 1 and 2) and *E. multilocularis* (Studies 3 and 4). Studies 2 and 4 also included treatment groups that received praziquantel alone or lotilaner alone, at the same minimum dosages, to assess whether lotilaner, moxidectin or pyrantel interfered with the activity of praziquantel in Credelio Quattro against these two cestode species. In Studies 1 and 3, 16 purpose-bred dogs were divided into two groups of eight, while in Studies 2 and 4, 32 dogs were divided into four groups of eight. Dogs were experimentally inoculated with protoscolices on Day – 28 for *E. granulosus* studies and on Day – 18 or – 20 for *E. multilocularis* studies. Credelio Quattro, placebo, praziquantel, or lotilaner tablets were administered orally at the lower half of the dosage range on Day 0. Efficacy was calculated based on the number of worms recovered at necropsy, 4 or 5 days post-treatment, in the treated group compared to the control.

**Results:**

Credelio Quattro was well tolerated in all dogs. Based on geometric mean worm counts, Credelio Quattro was 100% effective against both *E. granulosus* and *E. multilocularis.*

**Conclusions:**

Credelio Quattro administered once orally at the minimum dosages of 20 mg/kg of lotilaner, 0.02 mg/kg of moxidectin, 5.0 mg/kg of praziquantel and 5.0 mg/kg of pyrantel was safe and effective for the treatment and control of adult *E. granulosus* and *E. multilocularis* in dogs.

**Graphical Abstract:**

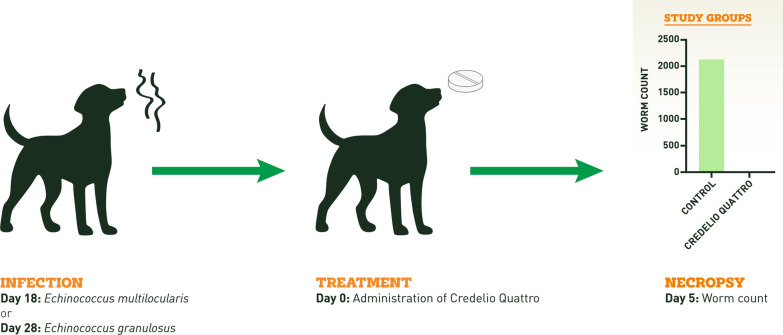

## Background

*Echinococcus granulosus* and *E. multilocularis*, small cestodes belonging to the *Echinococcus* genus in the Taeniidae family, are found worldwide [1], and the life cycle of these *Echinococcus* species relies on predator-prey relationships that involve two mammalian hosts. Wild and domestic canids serve as the definitive hosts for both *E. granulosus* and* E. multilocularis* [2]. The adult parasite lives in the small intestine of the definitive host, and the definitive host sheds eggs in their feces, which become embryonated and immediately infective [3]. Ungulates, typically sheep, moose and caribou, serve as intermediate hosts for *E. granulosus*, while rodents, including voles and lemmings, commonly serve as intermediate hosts for *E. multilocularis* [4]. When a suitable intermediate host ingests the infective embryonated eggs, the oncospheres hatch and migrate via the blood and lymphatic vessels into organs where they develop to the metacestode stage [5]. In the case of *E. multilocularis*, the liver is the primary organ affected. *Echinococcus granulosus* primarily affects the liver or lungs [5]. Metacestodes in other locations have been described for both cestodes [5]. The metacestode of *E. granulosus* develops as an unilocular hydatid cyst with expanding growth, while *E. multilocularis* develops as an alveolar hydatid cyst, with invading, infiltrative growth [4].

Humans, while not part of the natural life cycle, can inadvertently ingest eggs, leading to the development of alveolar echinococcosis due to *E. multilocularis* or cystic echinococcosis due to *E. granulosus*, severe and potentially fatal diseases primarily affecting the liver and lungs [5]. Though dogs are definitive hosts for *E. granulosus* and *E. multilocularis* infections, in rare cases, they can become infected with the metacestode stage of the parasite and experience alveolar echinococcosis disease [5, 6]. The World Health Organization (WHO) has listed echinococcosis as one of the 17 neglected diseases targeted for control or elimination by 2050; it estimates an incidence of cystic echinococcosis from < 1 to > 200 per 100,000 in specific rural populations and of alveolar echinococcosis from < 0.5 to > 100 per 100,000 in certain communities [7]. The control of *E. granulosus* and *E. multilocularis* infection in dogs is, therefore, a decisive measure in preventing echinococcosis in humans, wildlife and livestock [8].

Implementing an efficient and routine deworming regimen is crucial for controlling environmental contamination and significantly reducing the risk of these parasites spreading and transmitting to other dogs, intermediate hosts and humans [9, 10]. Here, we present studies on a new oral chewable tablet formulation (Credelio Quattro) that combines lotilaner, moxidectin, praziquantel and pyrantel, providing effective prevention of heartworm and lungworm disease, treatment and control of roundworm, hookworm and tapeworm infections as well as treatment and prevention or control of flea and tick infestations. Praziquantel, well established for anthelmintic efficacy against cestodes [11–13], was included in this combination tablet at the minimum effective dosage of 5 mg/kg.

## Methods

Four placebo-controlled, masked and completely randomized dose confirmation studies were conducted to evaluate efficacy. Two of these studies also served as non-interference studies. Studies 1 and 2 evaluated efficacy against *E. granulosus*. Studies 3 and 4 evaluated efficacy against *E. multilocularis.* Studies 2 and 4 included an investigation of whether the combination of individual active components interfered with the efficacy of praziquantel against *E. granulosus* or *E. multilocularis* and included groups treated with praziquantel or lotilaner alone.

All studies complied with VICH GL9, “Good Clinical Practice” [14], and were conducted consistent with VICH GL7, “Efficacy of Anthelmintics: General Requirements” [15], VICH GL19, “Effectiveness of Anthelmintics: Specific Recommendations for Canine” [16] and the World Association for the Advancement of Veterinary Parasitology (WAAVP): Second edition of guidelines for evaluating the efficacy of anthelmintics for dogs and cats [17].

### Animals

Purpose bred Beagle dogs in good health as confirmed by physical examination by a veterinarian were selected for enrollment into the studies.

For all dogs, no parasite eggs were seen on qualitative fecal examination, and no exposure to anthelmintics or endectocides containing a cestocide occurred within 27–35 days prior to enrollment. At experimental inoculation, dogs ranged in age from 6 to 10 months in three of the studies (Studies 1, 2 and 4) and 12–23 months in Study 3. Dogs ranged in weight from 6.5 to 15.9 kg at the time of treatment. Dogs received an age-appropriate commercial canine diet and access to potable water ad libitum. Dogs were housed in pens that conformed to animal welfare standards. After randomization, dogs were housed individually for the duration of the study. Health observations were conducted at least once daily on all dogs for the duration of the study.

### Study design and *Echinococcus granulosus* and *E. multilocularis* inoculation

In preparation for the studies, dogs were orally inoculated with infective protoscolices. In Studies 1 and 2, dogs received 10,000 or 11,400 *E. granulosus* (MT, USA) protoscolices on Day – 28, respectively. In Study 3, dogs were inoculated with 100,000 *E. multilocularis* (CH, EU) protoscolices on Day – 18 and in Study 4 dogs received 20,000 or 21,750 *E. multilocularis* (MT, USA) protoscolices on Day – 20. Inoculum size was based on recommendations in established guidelines [16, 17], regulatory compliance, historical expert experience working with these parasites, applicable publications [3] and availability of infective material collected from naturally infected animals in the field.

Dogs were allocated to treatment group (8 dogs/group) and individual housing on Day – 2 (Study 3) or Day – 1 (Studies 1, 2 and 4) in a completely randomized design. Treatment was on Day 0, and cestodes present in the gastrointestinal tract were recovered on Day 4 (Study 3) or Day 5. Tables 1 and 2 summarize the study designs and treatment groups.

**Table 1:** Summary of study designs depicting the day of inoculation, day of treatment and cestode recovery

Study	Parasite (origin)	Day of inoculation	Day of treatment	Day(s) of cestode recovery
1	*Echinococcus granulosus* (USA)	−28	0	5
2	*E. granulosus* (USA)	−28	0	5
3	*E. multilocularis* (EU)	−18	0	4 and 5
4	*E. multilocularis* (USA)	−20	0	5

**Table 2:** Summary of treatment groups depicting the number of animals in each group by study

Study	Parasite (origin)	Negative control dogs/group	Credelio quattro dogs/group	Praziquantel dogs/group	Lotilaner dogs/group
1	*Echinococcus granulosus* (USA)	8	8	n/a	n/a
2	*E. granulosus* (USA)	8	8	8	8
3	*E. multilocularis* (EU)	8	8	n/a	n/a
4	*E. multilocularis* (USA)	8	8	8	8

### Treatment

On Day 0, in the fed state, dogs were orally administered one of the following: placebo tablets (negative control), flavored chewable combination tablets (Credelio Quattro), lotilaner tablets (Credelio^™^, Elanco Animal Health) or praziquantel tablets (Droncit^®^, Elanco Animal Health), according to the study design. Dogs received the appropriate combination of tablets to provide as close to the lower half of the recommended dosage ranges (lotilaner 20–30 mg/kg, moxidectin 0.02–0.03 mg/kg, praziquantel 5.0–7.5 mg/kg, pyrantel [as a pamoate salt, 5.0–7.5 mg/kg]) as possible in each treatment group.

### Worm recovery

At necropsy on Day 4 or 5, the small intestine was isolated, removed and transferred to a 2-L container holding ~ 1 L tap water. The entire length of the small intestine was opened by sharp dissection. The entire mucosal surface was scraped lightly to remove contents, adherent mucus and parasites attached to the mucosa. Scrapings were combined with the contents of the 2-L container. Luminal contents, water rinses and scrapings were washed over a #100 (150 µm aperture) sieve which retained adult *E. granulosus* or *E. multilocularis*, including scolices; an individual sieve was assigned to each dog for worm collection. Material kept on the sieve was transferred to a labeled container (contents plus scrapings) and preserved with 10% formalin. The processed small intestine was placed in a labeled container, covered with 0.9% NaCL and allowed to soak for 2 to 4 h in an incubator at approximately 100 °F (37.8 °C). After incubation, the soaked intestine was stripped and the soak fluids and mucosal strippings again passed through the individual dog’s #100 sieve as previously described. Material retained on the sieve was transferred to a new labeled container (soaks) and preserved with 10% formalin.

From each dog, both samples, contents plus scrapings and soaks were enumerated separately in consecutive fashion using the same procedure: From each sample, two 5% aliquots were collected by volume and transferred to petri dishes for initial examination. The contents of each petri dish were scanned with a stereoscopic microscope at a total magnification of 10× to 30×. Worm counts were determined by registering each visualized specimen with a tally counter, with fragments only counted if the scolex was present. If ≥ 5 specimens were recovered during the initial examination from either aliquot of the sample, the total for both 5% aliquots was summed and multiplied by a factor of 10 to determine the total worm count for that sample. For samples with no or few (<5) worms found in the first two 5% aliquots, an additional 10% aliquot was examined with subsequent 20% aliquots examined until at least five worms were found or until the entire (100%) sample was evaluated for the absence of worms. The final worm count for each dog was determined by the worm count results of each sample (contents plus scrapings and soaks) and the percentage of each sample evaluated (10–100%).

### Statistical analysis

Efficacy of the novel combination tablet Credelio Quattro against experimentally induced *E. granulosus* and *E. multilocularis* infections was determined post-treatment by comparison of the number of adult cestodes recovered at necropsy in the Credelio Quattro group versus the control group. A minimum of five worms needed to be present in each of at least six control dogs to show an adequate infection in the control group [16, 17]. An efficacy of 100% for *E. granulosus* or *E. multilocularis* in the Credelio Quattro group and a demonstration of adequate infection in the control group were required to show overall efficacy of the combination tablet. Since 100% efficacy and adequate infection were demonstrated throughout all studies, no models were fitted to assess the effect of the combination tablet compared to the control group. Efficacy of 100% in Credelio Quattro group with efficacy of < 100% in the lotilaner group was required to conclude that praziquantel is the active ingredient in Credelio Quattro responsible for efficacy against cestodes. Percent efficacy (% Efficacy) was calculated as

$$\% \_{\text{Efficacy}}\, = \,{1}00\, \times \,({\text{GM}_{CTL}}{-}{\text{ GM}_{TRT}})/{\text{GM}_{CTL}}$$

where GM_CTL_ and GM_TRT_ represent the geometric mean worm counts for the control and treatment groups, respectively. Geometric means (GM) were calculated by back-transforming group arithmetic means of the log-transformed counts.

For each treatment group, the arithmetic means (AM) were additionally reported for all post-treatment worm counts.

## Results

No serious adverse events or mortality were seen in these studies. Non-serious, transient gastrointestinal signs were seen in all groups including control.

Table 3 summarizes the findings from these four studies, including total worm counts and percentage efficacy against each target parasite. In all four studies, eight of eight dogs in the control group had *E. granulosus* or *E. multilocularis* cestodes present at necropsy. All control dogs had worm burdens ≥ 5 worms per dog and thus demonstrated adequate infections.

**Table 3:** Arithmetic and geometric mean worm counts and percent efficacy on Day 4 or 5 post-treatment

Parasite assessed	Study	Arithmetic mean Mean (range)				Geometric mean Mean (% efficacy)			
		Group 1 (Control)	Group 2 (Credelio Quattro)	Group 3 (praziquantel)	Group 4 (lotilaner)	Group 1 (Control)	Group 2 (Credelio Quattro)	Group 3 (praziquantel)	Group 4 (lotilaner)
*Echinococcus granulosus*	1	1642.5 (930–2530)	0	NA	NA	1543.0	0.0 (100)	NA	NA
	2	2243.8 (1210–3830)	0	0	1816.3 (500–3910)	2118.4	0.0 (100)	0.0 (100)	1491.9 (29.6)
*E. multilocularis*	3	12,048.1 (1330–28,750)	0	NA	NA	6871.9	0.0 (100)	NA	NA
	4	442.5 (290–880)	0	0	510.9 (117–910)	413.8	0.0 (100)	0.0 (100)	418.7 (0)

### Efficacy against *Echinococcus granulosus*

In Study 1, all dogs in the control group had an adequate infection with cestode counts ranging from 930 to 2530 with an AM of 1642.5 and a GM of 1543.0. In comparison, all eight dogs that received Credelio Quattro tablet had zero cestodes, demonstrating 100% efficacy.

In Study 2, all dogs in the control group had an adequate infection with cestode counts ranging from 1210 to 3830 with an AM of 2243.8 and a GM of 2118.4. In comparison, all eight dogs that received Credelio Quattro tablets had zero cestodes, demonstrating 100% efficacy. In addition, all eight dogs that received praziquantel only had zero cestodes, whereas all eight dogs that received lotilaner only had cestode counts ranging from 500 to 3,910 (29.6% efficacy).

### Efficacy against *Echinococcus multilocularis*

In Study 3, all dogs in the control group had an adequate infection with cestode counts ranging from 1330 to 28,750 with an AM of 12,048.1 and a GM of 6871.9. In comparison, all eight dogs that received Credelio Quattro tablet had zero cestodes, demonstrating 100% efficacy.

In Study 4, all eight dogs in the control group had an adequate infection with cestode counts ranging from 290 to 880 with an AM of 442.5 and a GM of 413.8. In comparison, all eight dogs that received Credelio Quattro or praziquantel only had zero cestodes demonstrating 100% efficacy. Dogs that received lotilaner only had cestode counts ranging from 117 to 910 (0% efficacy).

## Discussion

No serious adverse events or mortalities were seen in any of the four studies. Non-serious transient gastrointestinal signs, like what is reported for other combination parasiticide products, were seen in all groups including control.

The efficacy of Credelio Quattro was evaluated in dogs with induced *E. granulosus* or *E. multilocularis* infections. The cestocide praziquantel, at the well-established and highly effective minimum dosage of 5 mg/kg, provides the cestode efficacy in Credelio Quattro. Praziquantel is known to have a wide therapeutic index. The results of the two non-interference studies presented here clearly demonstrate that praziquantel provided the cestode efficacy, and the presence of lotilaner, moxidectin and pyrantel pamoate in Credelio Quattro did not negatively affect the efficacy against either *E. granulosus* or *E. multilocularis*. A single oral administration of Credelio Quattro at the lower half of the effective dosage range, 5.0–7.5 mg/kg praziquantel, resulted in 100% reduction of adult *E. granulosus* or *E. multilocularis*.

Conducting a study with *E. granulosus* or *E. multilocularis* poses significant logistical challenges because of the nature of the parasite: highly zoonotic, small in size (1.2–7 mm) and unlikely detectable by the naked eye [18]. Due to the small size of *Echinococcus* cestodes, assigning an individual sieve to each dog was implemented based on the experience in an earlier development study to evaluate efficacy against *E. multilocularis*. In that study, dogs were administered a combination tablet containing lotilaner, moxidectin and praziquantel (5.0–7.5 mg/kg), without pyrantel, using a similar study design as described above (data not presented). After sieving the intestinal soak material of one dog administered the combination tablet, three worms were collected, although no worms were collected from the intestinal contents plus scrapings sample. The three worms found in the intestinal soak were attributed to cross-contamination; a control dog with a high worm count was processed just prior to the treated dog using the same sieve. Despite the cleaning procedure of the sieves between animals, inadvertent sieve contamination was suspected. All studies evaluating Credelio Quattro against *E. granulosus* and *E. multilocularis* included assigning an individual sieve to each dog for the processing and collection of worms. This refinement to the procedure aimed to exclude the possibility of cross-contamination. Praziquantel, whether administered alone or in Credelio Quattro, achieved 100% effectiveness in the treatment and control of *E. multilocularis* at the minimum effective dosage of 5 mg/kg.

As a definitive host of *E. granulosus* and *E. multilocularis*, infected dogs contaminate the environment with infective eggs. The control of infection in dogs is therefore important in preventing transmission of cystic and alveolar hydatid disease to humans, intermediate wildlife hosts and livestock [8].

A key element in treatment and control of intestinal cestode infections in dogs is pet owner compliance and routine and consistent strategic deworming of their dogs. Several veterinary parasitology organizations, including the Companion Animal Parasite Council (CAPC), American Heartworm Society (AHS), the European Scientific Counsel Companion Animal Parasites (ESCAPP) and the Tropical Council for Companion Animal Parasites (TroCCAP), provide evidence-based guidelines for parasite control. These guidelines emphasize the value of routine preventive veterinary care through physical examination and history, vector borne disease testing, microscopic fecal examination and/or advanced fecal diagnostic technologies to assess existing parasite infections and risk to parasite exposure. Veterinarians should make recommendations to their clients based on these established guidelines whenever possible. Credelio Quattro is designed with broad parasiticide activity against cestodes as well as heartworm, lungworm, roundworms, hookworms and fleas and ticks to enhance pet owner compliance through a single convenient monthly oral tablet.

## Conclusions

Credelio Quattro administered once orally (at the minimum dosages of 20 mg/kg of lotilaner, 0.02 mg/kg of moxidectin, 5.0 mg/kg of praziquantel and 5.0 mg/kg of pyrantel) was safe and effective for the treatment and control of adult *E. granulosus* and *E. multilocularis* in dogs.

## Data Availability

Data supporting the conclusions of this article have been presented within the article.

## Abbreviations

CAPC: Companion Animal Parasite Council
ESCCAP: European Scientific Counsel Companion Animal Parasites
TroCCAP: Tropical Council for Companion Animal Parasites
AM: Arithmetic Mean
GM: Geometric Mean
MT: Montana
USA: United States
CH: Switzerland
EU: Europe
WHO: World Health Organization
